# Culturally Tailored Tele–Mental Health Care Linkage for Indigenous Populations: Protocol for a Mixed Methods Pilot Study

**DOI:** 10.2196/67757

**Published:** 2025-11-12

**Authors:** Ariel M S Richer, Ariel L Roddy, Sutton King, Austin Serio

**Affiliations:** 1 College of Social Work The University of Utah Salt Lake City, UT United States; 2 Department of Criminology and Criminal Justice Northern Arizona University Flagstaff, AZ United States; 3 ShockTalk New York City, NY United States

**Keywords:** Native American, Indigenous, tele–mental health, telehealth, linkage-to-care, culturally tailored, depression, anxiety, substance use disorder, mental health, posttraumatic stress disorder, PTSD, qualitative interviews, artificial intelligence, AI, conceptual posttraumatic framework, social media, public health

## Abstract

**Background:**

Urban Indigenous populations face disproportionate mental health challenges, including high rates of posttraumatic stress disorder, depression, and substance use disorders, yet they have limited access to health services, especially culturally relevant care. The mechanism for providing care to Indigenous people in the United States, the Indian Health Service, is significantly underfunded and only accessible to certain Indigenous people. With more than 70% of Indigenous individuals in the United States living in urban settings, there is a growing need for innovative health care solutions. A community-based, Indigenous-led health and mental health–focused nonprofit in the northeast United States developed ShockTalk, a tele–mental health linkage-to-care app tailored specifically for Indigenous communities, to fill this gap.

**Objective:**

This study aims to assess (1) the feasibility, including accessibility, experience, and value, of ShockTalk for practitioners and clients interested in providing or receiving culturally responsive mental health treatment, and (2) changes in client attitudes related to tele–mental health treatment value and trust in ShockTalk technology.

**Methods:**

This study outlines the development and pilot study of ShockTalk. The conceptual framework is based on the behavioral model of health care use. ShockTalk uses artificial intelligence to connect clients with Indigenous or culturally aligned therapists and facilitates access to care via Facebook Messenger. Using a prewaitlist or postwaitlist design, 5 client participants will be admitted to the study at first, and 5 additional participants at 3 months. Data collection includes presurveys and postsurveys on client attitudes toward mental health treatment and trust in the ShockTalk platform at baseline and a 3-month follow-up, followed by in-depth qualitative interviews at 3 months. A preliminary economic evaluation will track direct costs (ie, therapist time, platform fees, and administrative expenses) and compare relative costs across treatment doses. Analyses will assess the feasibility of data collection and inform a future full-scale trial.

**Results:**

This study was funded in April 2022. Data collection occurred between May 2022 and October 2024. In total, 4 client participants and 2 therapist participants were enrolled. Data analysis is complete and results are expected to be published in February 2026.

**Conclusions:**

This pilot study will offer insights into optimizing technology-based, culturally relevant mental health care. By examining varying levels of engagement and associated costs, this research seeks to identify the most effective and cost-efficient strategies for improving mental health outcomes in urban Indigenous populations in the United States. ShockTalk has the potential to shape future health care innovations in this field. Findings are expected to contribute significantly to Indigenous mental health care by offering insights into sustainable, accessible, and culturally appropriate telehealth interventions, guiding future policy and practice.

**International Registered Report Identifier (IRRID):**

DERR1-10.2196/67757

## Introduction

### Background

We use the term *Indigenous*, which does not center on arbitrary geopolitical lines, to describe American Indian, Native American, and Alaskan Native populations in what is now known as the United States. Urban-living Indigenous people experience disproportionately higher rates of mental health disorders [1,2], often as a result of forced isolation from their homelands of origin, communities, and a lack of adequate mental health care resources [3,4]. The most significant mental health concerns facing urban Indigenous communities include a high prevalence of substance use disorders [5], depression, suicide, anxiety [6], and posttraumatic stress disorder (PTSD) [2]. For example, estimates of rates of PTSD among Indigenous people range from 16% to 24% [7-9], which is 3 times the rate of lifetime PTSD among the general population [10], and rivals the lifetime rate (29%) among US veterans returning from Iraq and Afghanistan [11].

### Access to Mental Health Services

Addressing the mental health disparities among Indigenous communities that reside in urban contexts presents unique challenges. The Indian Health Service (IHS), created in 1955 through Public Law 568, The Transfer Act of 1954, exists to fulfill the US government’s treaty obligations and legal responsibilities to provide health care to Native American and Alaska Native tribes [12]. The IHS is currently funded at less than half of its required budget [13] and faces significant challenges, including barriers to service, staffing shortages, extended wait times, and diminished access to quality care [14]. Notably, the IHS dedicates only 1% to 2% of its line budget to urban Indian health programs, which are funded only through contracts, and many of these programs have limited capacity [15]. Due to colonial policies, such as the termination, boarding school, and relocation eras, many Indigenous community members have been displaced from their original homelands into urban areas [16]. As a result, more than 70% of Indigenous people, specifically those identified as American Indian or Alaska Native, in the United States today live in urban areas [17], of which only 25% live within an IHS service area [15]. The discrepancy between the location of Indigenous medical services and the areas in which most Indigenous individuals in the United States live poses a significant challenge, highlighting the need for health care innovation.

Furthermore, the rates of health insurance coverage are markedly lower for Indigenous people compared with those for non-Hispanic White individuals, compounding this health disparity. As of 2019, just over half (51.9%) of Indigenous individuals alone or in combination had private health insurance coverage [6]. The percentage of Indigenous people who were enrolled in Medicaid or other public health insurance coverage was 42.9%, and 14.9% of Indigenous individuals did not have health insurance coverage; the comparative rates for non-Hispanic White individuals were 74.7%, 34.3%, and 6.3%, respectively [6]. Therefore, there is a profound need to improve and expand systems of care to help urban Indigenous individuals access affordable mental health treatment.

### Telehealth and Indigenous Populations

Telehealth, or telemedicine, uses two-way, real-time telecommunication audio and video technologies, such as computers, tablets, or smartphones, to deliver health care services and public health [18]. Telehealth interventions allow health care providers to meet with patients remotely to diagnose, consult, treat, educate, and manage care, and they also allow patients to manage their care [19]. Implementing care and interventions among underserved populations through telehealth has been shown to be feasible and cost-effective [20], with the ability to be culturally tailored to meet the needs of Indigenous populations [21]. In a recent scoping review regarding telehealth use by Indigenous populations from Australia, Canada, New Zealand, and the United States [21], 47% of the 321 studies identified used mobile health (mHealth), and 26% focused on mental health. Indigenous communities expressed concerns regarding privacy and confidentiality, limited broadband internet, uncertainty in their ability to use the technology, and lack of trust in the therapeutic relationship. However, collaboration with Indigenous communities to develop and implement the intervention and offer Indigenous health care providers or cultural liaisons (13%) in its delivery indicated an increase in cultural safety. Notably, none of the identified studies discussed interventions that served solely as a linkage to mental health care or facilitated a way for Indigenous clients to identify mental health support.

With these goals in mind, this manuscript provides a detailed protocol for the feasibility pilot of the first tele–mental health linkage-to-care app tailored specifically for Indigenous communities, named ShockTalk. The purpose of the telehealth app is to improve access to culturally relevant mental health care for Indigenous individuals. The following section outlines the conceptual framework, the process of development for the mobile app, and identifies the needs of community members.

### Conceptual Framework

The proposed research is informed by the behavioral model of health care use [22], which aims to understand how health care use and health-related outcomes are influenced by predisposing, enabling or inhibiting, and need factors at both the individual and contextual (eg, community and policy level) characteristic levels. Individual and contextual factors also influence personal health practices and care processes. In an iterative cycle, outcomes affect subsequent predisposing and enabling factors, the perceived need for health services, and health behavior.

**Figure 1:**
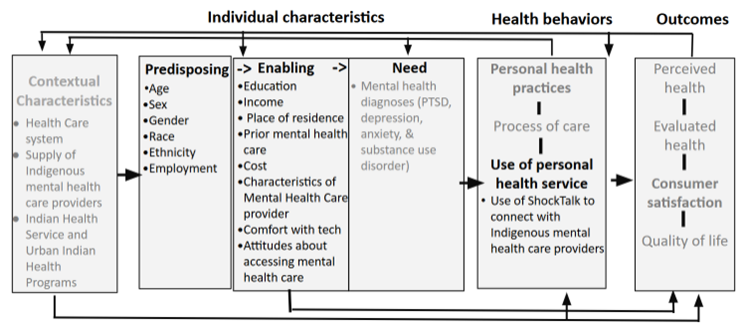
Conceptual framework for the ShockTalk pilot study. PTSD: posttraumatic stress disorder.

Figure 1 depicts the conceptual framework of this study. The variables shown in black font will be included in our preuse and postuse survey. Guided by these findings, primary data collection and analysis of in-depth interviews will seek to better understand the components of the conceptual model in gray font (eg, process of medical care). This study focuses on the relationship between *individual characteristics* and subsequent *health behaviors*.

### Preliminary ShockTalk App Development

ShockTalk was conceptualized by 3 New York City–based Indigenous individuals with experience in social work, public health, technology, and research. To better understand the barriers to and facilitators of culturally responsive mental health service access in urban areas for Indigenous individuals, 70 in-depth interviews with self-identified Indigenous individuals were conducted by the community-based partner team who originally conceptualized this app with community members and mental health clinicians in the urban area of Lenapehoking (specifically, in what is now known as New York City). The findings of this assessment are presented in Table 1. Finances, lack of insurance, and lack of systems to help Indigenous clients identify culturally appropriate service providers were identified as the most prominent barriers through qualitative analyses. Facilitators of care included sliding-scale service offerings and connections to Indigenous service providers or health care providers that match the client on other statuses or identities.

**Table 1:** Themes from the preliminary ShockTalk development.

Theme	Findings
Structural barriers to accessing therapy	Perceived stigma of accessing therapy Need for variety of therapeutic settings, online, in person, and “meet the client where they are” Lack of availability of Indigenous therapists Access to therapy, including telehealth Limited scheduling and availability, and a cumbersome process
Interpersonal barriers to accessing therapy	Lack of comfort with the therapist Uncertainty about online therapy Fear of not having a cultural connection Cost Lack of spiritual component in Western-dominant therapy
Characteristics of an ideal therapist	Ability to connect deeply with someone “beyond the surface” who is “down to earth” Relational (ie, compassionate and lets the person talk) vs advice giving Can assure privacy and trust, especially in a small community Shared characteristics and understanding of experience Shared understanding of trauma and colonization Understanding of Indigenous culture, experiences, and ways of knowing Share Native or Indigenous identity who better understand the “everyday struggle” connected with Indigenous identity History of working with Indigenous people “in a good way” Feel connected Can offer cultural or spiritual coping mechanisms and tools

### ShockTalk App: How It Works

**Figure 2:**
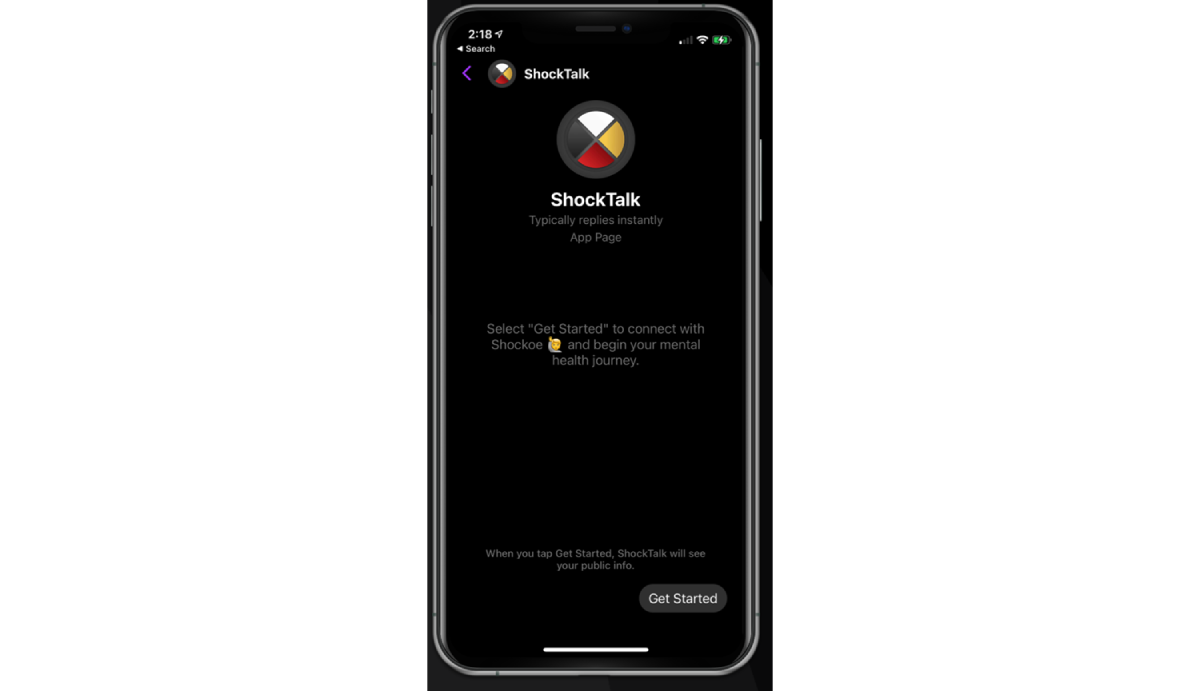
Preview of the ShockTalk tele–mental health linkage-to-care app.

ShockTalk (Figure 2) is a mobile app that uses Facebook Messenger as its entry point for clients. When a client first connects, they are greeted by ShockTalk’s artificial intelligence (AI) assistant, which guides them through the initial process. The AI collects basic contact information, helps synchronize important dates directly to the client’s calendar, and offers an option to schedule a 15-minute intake session with a licensed therapist from ShockTalk’s vetted health care provider directory.

Before the intake appointment, the AI sends reminder messages to help clients prepare. During the intake, the therapist and client discuss needs, goals, and preferences. Together, they decide whether the therapist is a good fit for ongoing care. If so, they begin a therapeutic relationship within the platform. If not, the therapist helps connect the client to other appropriate services or health care providers.

### Objective

The ShockTalk team has partnered with a researcher affiliated with the Columbia University School of Social Work to evaluate the pilot and feasibility of this app, which will involve interviews with 10 Indigenous mental health treatment seekers in Lenapehoking and 3 practitioners. The aims of this research are as follows: (1) assess changes in client attitudes related to tele–mental health treatment value and trust in ShockTalk technology; (2) assess the accessibility, experience, and value of ShockTalk for practitioners and clients interested in providing or receiving culturally responsive mental health treatment; and (3) estimate the potential benefit of the culturally responsive mental health treatment facilitated by ShockTalk. The ultimate evaluation of this pilot program will assess the prospective benefits of tele–mental health care linkage for Indigenous individuals living in urban settings.

## Methods

### Overview

To test the feasibility of ShockTalk among Indigenous clients, we will conduct a small pilot study with 10 Indigenous therapy-seeking clients and 3 Indigenous mental health practitioners in Lenapehoking. This study was designed as a preliminary feasibility pilot to inform a larger trial and, therefore, was not prospectively registered.

After informed consent and baseline assessment, clients will be randomly allocated 1:1 to immediate access to ShockTalk (treatment) or to a 3-month waitlist control (5 clients per arm). The allocation list will be generated by a study team member not involved in enrollment and uploaded to a centralized randomization module, which will reveal the assignment only after baseline data entry. Allocation events will be logged. In the absence of a centralized system, sequentially numbered, sealed opaque envelopes prepared from the same allocation list will be used.

All participants will complete baseline surveys and a 3-month follow-up survey (3 months postrandomization). Waitlist participants will be offered access to the linkage-to-care intervention 3 months after their initial baseline survey is completed. Qualitative data collection at 3 months from baseline will include 1-hour semistructured interviews (Multimedia Appendix 1) with a purposive subsample of 5 clients (selected to reflect a range of engagement and experiences across arms) and a single focus group with participating practitioners. Finally, a preliminary cost analysis will be conducted using data from both arms to assess the feasibility of cost data collection and to generate exploratory cost estimates across varying levels of engagement. Analyses will be descriptive and feasibility-focused (ie, recruitment, retention, acceptability, and data completeness); inferential effectiveness claims will not be made given the pilot sample size.

### Study Sample Recruitment

Ten Indigenous clients and 3 Indigenous practitioners will be recruited to assess the feasibility and acceptability of ShockTalk on care linkage among therapists and clients. This proposed sample size aligns with other pilot and feasibility studies, which are not powered for hypothesis testing and are instead based on the pragmatics of recruitment [23]. Therapists will be contacted directly via email from a listserv that was previously compiled through the ShockTalk company website. Therapists who signed up with their names and email addresses and consented to being contacted later will be contacted by our team about their continued interest in ShockTalk and whether they would participate in the pilot program of the app. Indigenous therapy-seeking participants will be recruited via the ShockTalk internal listserv and social media posts on Facebook, Instagram, and X (formerly Twitter), which will include brief information about the study, compensation, and participant expectations.

### Inclusion and Exclusion Criteria

Participants will be screened for the following eligibility criteria: (1) are aged at least 18 years, (2) self-identify as Indigenous to North, South, Central Americas, or the Caribbean, and (3) live in New York State. Individuals are not eligible if they live outside of New York State, do not self-identify as Indigenous, or are less than 18 years of age. Practitioners will be screened for the following eligibility criteria: (1) are a mental health professional licensed to practice in New York State and (2) self-identify as Indigenous to North, South, or Central America or the Caribbean.

### Data Safety and Monitoring

The study facilitator, under the supervision of the principal investigator, will monitor data. Data will be securely stored on a password-protected computer, laptop, and on a password-protected Google Drive associated with the study facilitator’s university-administered email address. Hard-copy data will be stored in a securely locked filing cabinet in the study coordinator’s office, to which only the study facilitator will have access. All data collection will be entirely anonymous; no identifiers will be associated with the ID number labeled on the data.

### Potential Risks

Despite best efforts to protect privacy and confidentiality, participants face a risk of potential loss of confidentiality. During the health care provider focus group, there is a potential for loss of confidentiality, although all participants are asked to keep their involvement in the study and what is shared during the focus group private. During an in-depth interview, there may be risks associated with discussing potentially stressful subjects. There is no more than a minimal risk of any harm to participants.

### Potential Benefits

Participants may benefit from increased access to a culturally relevant mental health therapist. Some participants may find the in-depth interview process empowering and therapeutic in experience. Additionally, the information collected from this research may help others in the future.

### Ethical Considerations

This study has been approved by the Columbia University Institutional Review Board (AAAU0850). The study coordinator will contact client and practitioner participants to determine their eligibility and to discuss the study purpose, risks, benefits, and compensation. During this meeting, the study coordinator will also obtain informed consent. Participants will be provided with an electronic version of the consent form and will be asked to sign and return the form at the end of the call.

Participants will be informed that they can opt out at any point during the study. Data will be anonymized, and no names or other identifiable features of research participants or users will be disclosed in any manuscript or supplementary material.

Participants (therapy seekers) will be compensated US $10 for completing each survey, one at baseline and one at 3 months follow-up. Participants (therapy seekers) will receive US $30 for completing an in-depth interview. In total, participants are eligible to receive a total of US $50 for completing both surveys and the in-depth interview. Additionally, as a part of participating in this study, participant clients will have the cost of their therapeutic sessions covered, up to 5 sessions and no more than US $600. Therapist participants will be compensated with US $40 for completing the focus group session.

### Dissemination

Initial findings will be disseminated first to participants to obtain their feedback and to ensure that the themes that are developed from the qualitative interviews align with their experience and accurately represent their views. Additionally, results from this study will be disseminated in culturally relevant and accessible ways, such as community meetings, social media posts, and a community-facing report that will be developed with ShockTalk. Findings will be used to improve the current ShockTalk app and linkage-to-care process. The results will also be used to facilitate the search for additional sources of funding to implement broader assessments.

### Measures

#### Quantitative Measures

The first survey instrument will examine changes in attitudes related to tele–mental health treatment value for clients before and after treatment facilitated by ShockTalk. One month before treatment and 3 months after treatment, clients will be given the Attitudes Toward Seeking Professional Psychological Help-Short Form survey [24]. This survey measures participants’ openness to seeking professional help for emotional problems and the value and need for seeking professional help. Clients are asked whether they disagree, partly disagree, partly agree, or agree with an item, including questions such as, “The idea of talking about problems with a psychologist strikes me as a poor way to get rid of emotional conflicts” and “A person with an emotional problem is not likely to solve it alone; he or she is likely to solve it with professional help.” Their attitudes toward seeking professional help are scored based on the sum of all responses. The short version of the scale demonstrates internal consistency ranging between 0.82 and 0.84, and high test-retest reliability (a=0.80) [24]. Once the presurveys and postsurveys are collected, the study team will examine the differences in openness to treatment before and after ShockTalk*.* A positive difference between the pretreatment and posttreatment Attitudes Toward Seeking Professional Psychological Help-Short Form score will indicate an increase in client valuation of mental health care treatment. *t* tests (1-tailed) will be used to evaluate whether the differences are statistically significant.

The second survey addresses trust and accessibility in tele–mental health care services and linkage services for clients. Clients will be asked 5 items each from the Trust in Technology and Trust in Telemedicine Service scales from the Patient Trust Assessment Tool (PATAT) [25]. The PATAT is a trust benchmarking survey and, currently, the only instrument that provides a comprehensive measurement of trust in an eHealth service and the underlying trust concepts. Clients will respond to questions related to their trust in technology and telemedicine surveys using responses on a Likert scale, which ranges from totally disagree (1) to totally agree (5). Questions from the Trust in Technology subset include items such as “Everything done on ShockTalk remains private” and “The personal information that is stored at ShockTalk will not get lost.” Items from the Trust in Telemedicine Service include questions such as “I trust ShockTalk less than other online services.” In conjunction, these 2 quantitative surveys will assess practitioner and client trust in the ShockTalk app and changes in client valuation of treatment resulting from culturally appropriate telehealth care linkage.

#### Qualitative Measures

To identify potential service gaps or accessibility concerns in the ShockTalk app, we will conduct in-depth interviews with the practitioners and clients 3 months after treatment participation. Practitioners will be interviewed in a talking circle, a technique consistent with Indigenous methodologies [26]. In the talking circle, practitioners will be asked a series of open-ended questions related to their past experiences with telehealth care linkage systems and how ShockTalk compares with other systems in terms of accessibility, organization, and usability. They will also be asked about their favorite elements of the app, any problems they experienced, and potential improvements that might benefit both the client and the practitioner. The qualitative interview consists of 6 base questions with several probing questions. The talking circle is expected to take 1 to 2 hours.

Clients will also engage in one-on-one in-depth interviews. Although talking circles may be preferable to facilitate discussions, the sensitive nature of the client interviews prevents the study team from conducting discussions of this type. Clients will be asked about their previous experiences in therapeutic contexts, how their experience in therapy resulting from ShockTalk is similar to or different from those experiences, and what could be done to make the app more accessible. Clients are asked to walk through the process of accessing ShockTalk and discuss points at which the app was not intuitive or was problematic. After discussing the process and experience of using the app, clients will be asked about their process in choosing and retaining a therapist—what therapist attributes were most important in deciding on a therapist, what factors influenced a therapeutic fit, and what influenced their likelihood of rescheduling with the therapist they saw. Finally, clients are asked about any potential changes or improvements they would make to improve their experience. Each client interview is expected to take 30 minutes to 1 hour.

### Planned Analysis

#### Qualitative Analysis

To analyze the qualitative data, an exploratory approach within an applied thematic analysis framework will be used in this study. Following the 6 phases of thematic analysis of Braun and Clarke [27], the process will begin with familiarization through repeated readings of the interview transcripts and initial memo writing to capture early analytic impressions. In the next stage, initial codes will be generated inductively from the data. Codebooks will be developed separately for client and practitioner transcripts, allowing codes to reflect participant language and meaning while attending to the study’s focus on the accessibility, experience, and value of ShockTalk.

After coding, the research team will search for themes by collating related codes into broader categories that capture patterned responses across the dataset. These candidate themes will then be reviewed for coherence and consistency within and across transcripts. To enhance rigor, 2 researchers will independently code transcripts, and coding decisions and candidate themes will be refined collaboratively during team meetings.

In the defining and naming stage, themes will be further refined to ensure clear boundaries and internal coherence, and concise names and definitions will be developed to represent their essence. Finally, the reporting phase will involve weaving together the analytic narrative with illustrative quotations from participants to demonstrate how themes capture client and practitioner perspectives on ShockTalk’s accessibility, user experience, and value, and to link these findings back to the research objectives.

#### Cost Analysis

Given the pilot nature and limited sample size of this study (n=10), the economic evaluation is not designed to conduct a formal cost-effectiveness analysis. Instead, the cost analysis is focused on assessing the feasibility of cost data collection, identifying relevant cost components, and reporting preliminary, descriptive cost estimates across varying levels of engagement with the ShockTalk intervention.

We will adopt a health care provider perspective for this analysis, capturing the direct costs related to service delivery through the ShockTalk app. These costs will include (1) therapist compensation for up to 5 sessions per client, (2) platform use and administrative costs, and (3) personnel time involved in scheduling, coordination, and support. The time horizon for the analysis is 3 months, aligning with the study’s follow-up period.

Clients’ level of engagement will be characterized by treatment dose, defined as the number of therapy sessions completed during the 3-month period. Although we will descriptively report outcomes stratified by dose (eg, 1-2 sessions and 3-5 sessions), no inferential comparisons across groups will be made.

The primary outcome for exploratory effectiveness assessment in the cost analysis will be the change in client attitudes toward mental health treatment, as measured by the ATSPPH-SF. Secondary outcomes include trust in technology and telemedicine, measured via the PATAT, and client engagement, operationalized through session attendance and app interaction data.

This feasibility-focused cost analysis aims to determine the viability of collecting accurate and comprehensive cost and outcome data in a small-sample, community-engaged study context. Given the pilot nature of the study, these analyses are intended to test the feasibility of collecting cost data and to provide exploratory estimates to guide the design and analytic strategy for a future, fully powered economic evaluation.

#### Quantitative Analysis

Quantitative analysis will involve linear mixed-effects models to understand the impact of different treatment doses on mental health outcomes, accounting for repeated measures and potential intragroup correlations. ANOVA will be used to compare the mean outcomes across the dose groups. Because of sample size limitations, we acknowledge that *t* tests and ANOVA may be underpowered to detect the true impact of the intervention on changes in attitudes surrounding therapy. In tandem with the cost analysis, the ANOVA will provide insights into the optimal balance between treatment effectiveness and cost, guiding resource allocation and decision-making for culturally responsive mental health services delivered through the ShockTalk app.

## Results

This study was funded in April 2022. Data collection occurred between May 2022 and October 2024. During this period, 4 client participants and 2 therapist participants were enrolled. Data analysis is complete and results are expected to be published in February 2026.

## Discussion

### Anticipated Findings

Given the limitations of the service provisions of IHS to service Indigenous communities in the United States, there exists a pressing need to connect urban-living Indigenous people to mental health services. The purpose of this work is to assess the potential of ShockTalk, the first tele–mental health care linkage app created for Indigenous treatment seekers, to improve the landscape of treatment. This research is novel as there is no current assessment of this kind and is salient given the significant mental health concerns of this population. Overall, the findings of this work are expected to facilitate improved mental health care provisions for a population of exceptional need.

### Limitations and Future Research

Due to the nature of the pilot and feasibility aims and funding limitations, the outcomes of this work will be limited by the sample size. Specifically, it is not possible to establish statistically significant differences in outcomes before and after treatment with only 10 participants. However, this pilot study was intentionally sized to address feasibility objectives (recruitment yield, retention, acceptability, and protocol fidelity) and to produce actionable, community-driven improvements to the intervention and study procedures before a larger trial could be conducted. Practical constraints also informed the sample size: the project is community-partnered and prioritizes culturally appropriate engagement and data sovereignty, which limits rapid scale-up; the pilot grant resources were modest; and protecting participant confidentiality in a small, close-knit community was a priority. The findings from this pilot study will be used to refine measures, define progression criteria, and calculate sample sizes and power for a subsequent randomized trial. The results of this pilot program will be used to seek additional funding opportunities, which will be used to fund additional sites of research across the United States. Additional data collection will be performed to increase the statistical power for quantitative analysis and improve the generalizability of the results.

### Conclusions

The disproportionate burden of mental health disorders among urban Indigenous community members highlights the critical need for innovative and culturally responsive health care delivery systems. ShockTalk, as the first tele–mental health care linkage app designed specifically for Indigenous individuals, represents a promising intervention to bridge this gap. This pilot study’s comprehensive evaluation of ShockTalk will provide valuable insights into how culturally responsive, technology-mediated mental health treatments can be optimized and tailored to meet the unique needs of Indigenous clients in urban settings. By assessing different intensities of engagement and their associated costs, this research aims to identify the most effective and economically viable treatment strategies.

The findings from this study are poised to significantly contribute to the field of Indigenous tele–mental health care by demonstrating the potential benefits of integrating culturally relevant telehealth services into broader mental health support frameworks. By examining variations in treatment doses and their impact on client trust, engagement, and overall satisfaction, we aim to delineate the most effective approaches for delivering mental health care that is culturally appropriate and accessible to this population. The integration of a cost-effectiveness analysis further ensures that the proposed solutions are sustainable and scalable. Future expansions of this research, supported by additional funding, will extend these insights to a larger and more diverse subset of the urban Indigenous population, ultimately guiding policy and practice toward more equitable mental health care provisions.

## Appendix

Multimedia Appendix 1 Client and health care provider interview and focus group guide.

## Abbreviations

AI: artificial intelligence
IHS: Indian Health Service
PATAT: Patient Trust Assessment Tool
PTSD: posttraumatic stress disorder

## Data Availability

The datasets generated or analyzed during this study are not publicly available due to the small sample size and sensitive participant information but are available from the corresponding author upon reasonable request.
