# Supplementary material for: Culturally Tailored Tele–Mental Health Care Linkage for Indigenous Populations: Protocol for a Mixed Methods Pilot Study
Source: JMIR Res Protoc. 2025 Nov 12;14:e67757. doi: 10.2196/67757 (PMC12658390; doi:10.2196/67757)
Supplement: Multimedia Appendix 1 [file resprot_v14i1e67757_app1.docx]

**Client Participant Interview Guide**

1. What was your experience with therapy before this pilot program?
   1. In person, online?
   2. How has this been different or the same from experience or expectations?
2. Accessibility
   1. What would make ShockTalk more accessible?
3. Walk us through your process of accessing Shock Talk Beta
   1. Initial intake email
   2. Initial intake sessions
   3. Did you get the reminders and prompts? Were they helpful
   4. After your first intake, how did you schedule your next session? And the next?
   5. Did you schedule subsequent sessions through the app?
4. How did you choose the therapist? What was most important to you? (ex:Gender, sex, cultural identity?)
   1. How did you know it was a good fit?
   2. What would make you reschedule with this therapist? What would influence your decision to stay in their care?
5. What didn’t you like about ShockTalk App?
6. How would you improve this process?
7. What else would you like to add?

**Therapists Participant Interview Guide**

1. What other online apps/systems have you used to connect with clients?
   1. How does this compare with ShockTalk Beta App?
   2. What would you change?
   3. What would make this more accessible to other Indigenous and Native clients?
   4. What are some of the greatest barriers to accessibility?
2. What did you like about the ShockTalk App?
   1. What was the process of connecting your calendar to Calendly?
   2. Did you use ShockTalk to schedule future sessions with clients? Why or why not?
3. What didn’t you like about the ShockTalk App?
4. How would you improve this process?
5. Do you have any concerns using the ShockTalk Beta App?
6. Anything else that you would like to add?
